# Associations of dichlorophenol with metabolic syndrome based on multivariate-adjusted logistic regression: a U.S. nationwide population-based study 2003-2016

**DOI:** 10.1186/s12940-023-01037-z

**Published:** 2023-12-15

**Authors:** Jing Cai, Zhichao Yang, Sen Zhao, Xing Ke

**Affiliations:** Key Laboratory of Drug Prevention and Control Technology of Zhejiang Province, Zhejiang Police College, Hangzhou, 310053 P.R. China

**Keywords:** Para-dichlorobenzene, 2,5-dichlorophenol, Urine, Metabolic syndrome, Population-based study

## Abstract

**Background:**

Para-dichlorobenzene (p-DCB) exposure associated with oxidative stress has indeed raised public concerns. However, whether p-DCB is linked with metabolic syndrome (MetS) remains unclear. We hypothesized that higher exposure to p-DCB would be linked with a higher risk of MetS in the U.S population. This study aimed to examine the associations of exposure to p-DCB with MetS prevalence.

**Methods:**

We included 10,428 participants (5,084 men and 5,344 women), aged ≥ 20 years, from the National Health and Nutrition Examination Survey (2003–2016). The cases of MetS were diagnosed by NCEP/ATPIII. Logistic regression models were conducted to calculate the odds ratios (ORs) and 95% confidence intervals (CIs) of MetS prevalence. Moreover, the mix associations of p-DCB metabolites were assessed using quantile sum (WQS) regression and quantile g-computation (qgcomp) methods.

**Results:**

We documented 2,861 (27.1%) MetS cases. After adjustment for the potential risk factors, the ORs (95% CI) of MetS prevalence across the quartile of urinary 2,5-dichlorophenol (2,5-DCP) were 1.09 (0.93-1.28), 1.22 (1.00-1.49), and 1.34 (1.04-1.73). Moreover, 2,5 DCP is significantly associated with a higher prevalence of abdominal obesity [OR_Q4vsQ1_ (95% CI): 1.23 (1.03-1.48)]. The WQS and qgcomp index also showed significant associations between p-DCB metabolites and MetS. Moreover, we further examined that 2,5 DCP was correlated with higher systolic blood pressure (*r* = 0.022, *P* = 0.027), waist circumference (*r* = 0.099, *P* < 0.001), and glycohemoglobin (*r* = 0.027, *P* = 0.008) and a lower high density cholesterol (*r* = -0.059, *P* < 0.001). In addition, the significant positive associations between 2,5 DCP and MetS were robust in the subgroup and sensitivity analyses.

**Conclusion:**

These findings indicated that increased urinary p-DCB concentration, especially 2,5 DCP, had a higher MetS prevalence. These results should be interpreted cautiously and further research is warranted to validate our findings.

**Supplementary Information:**

The online version contains supplementary material available at 10.1186/s12940-023-01037-z.

## Background

Metabolic syndrome (MetS) is a group of risk factors that contain high blood pressure, hyperlipidemia, and disturbance of glucose homeostasis [1]. The prevalence of MetS has become an increasing public health burden, which affects about a quarter of adults worldwide [2]. Epidemiological evidence from the NHANES study shows that the prevalence of MetS has continued to rise and reached 34.7% in 2011-2012 due to the increased prevalence of overweight and obesity rates in adults [3, 4]. The development of MetS influences individual life and leads to further cardiometabolic disease, including cardiovascular disease (CVD) [5] and type 2 diabetes (T2D) [6]. Thus, it is warranted to prevent the deteriorating development of MetS. Although MetS was mainly impacted by dietary and lifestyle factors, such as excessive energy intake and lack of exercise [1], increasing evidence suggested that Environmental pesticides, such as Para-dichlorobenzene (p-DCB), also have the potential to increase MetS prevalence [7–9].

p-DCB, an organic compound, is poorly soluble in water and has been widely used as a disinfectant, pesticide, and deodorant [10, 11]. Previous studies found that p-DCB was correlated with other organochlorine compounds that have widespread existence in new buildings, restrooms, and the air of households but may also pose some potential risks to the environment and health [12]. The International Agency for Research on Cancer (IARC) suggested that p-DCB may reasonably be a carcinogen based on animal evidence [11]. People may be exposed to p-DCB in mothballs, toilet deodorizer blocks, and air fresheners [13]. After inhaling paradichlorobenzene, human volunteers exhaled half of the dose. One hour after exposure ceased, the concentration of paradichlorobenzene in their blood had dropped by over 50% [14]. 2,5-dichlorophenol (2,5-DCP) and 2,4-dichlorophenol (2,4-DCP) are the major metabolites of p-DCB [15, 16]. Given that 2,5-DCP is readily detectable at low concentrations, it is well suited for monitoring daily exposure to p-DCB [15]. NHANES study reported that 2,4-DCP and 2,5-DCP were found in 64% and 98% of U.S. adult urinary samples [17].

The global community has expressed significant concern regarding the potential health hazards posed by a combination of chemical exposure [18, 19]. A recent study identified the mixed chemicals were significantly associated with lipid profiles in Korean adults [18]. Nguyen et al. (2022) also found that cadmium, mercury, and lead had positive associations with liver enzymes and NAFLD indices [19]. There is growing evidence of the effects of p-DCB on noncommunicable diseases [7, 12]. Epidemiological studies reported that higher 2,5-DCP levels were related to a higher obesity prevalence [20]. Another cross-sectional study from U.S adults also determined the positive association between 2,5-dichlorophenol and diabetes [12]. Emerging evidence found 2,5-DCP and 2,4-DCP were positively associated with the prevalence of hypertriglyceridemia in Mexican women [ORs (95% CI): 1.74 (0.98-3.05) for 2,5-DCP and 1.78 (0.99-3.23) for 2,4-DCP] [8]. These studies suggested that p-DCB and its metabolites are linked with the risk factors of MetS. Although the previous study showed a positive association of 2,5-DCP with MetS prevalence among non-diabetic adults [7], the small size sample (*n* = 1,706) and insufficient assessment (lacking sensitivity analysis) make it difficult to account for the robustness of the results. Moreover, the association between p-DCB exposure and MetS among the general population remains unclear. Meanwhile, no study has looked into the relationship between a combination of p-DCB exposure and MetS in US adults.

To address the above-mentioned knowledge gaps, this study aims to evaluate the associations of internal exposures to p-DCB with MetS prevalence from the NHANES (2003-2016) among 10,428 participants. We also conducted subgroup and sensitivity analyses to verify the robustness of the results.

## Methods

### Study participants

NHANES is a national representation, multi-year cycle, multi-stage sample design, and cross-sectional study among the US noninstitutionalized civilian [21]. In this study, we aggregated data from seven survey periods on p-DCB from seven cycles, including 2003-2004, 2005-2006, 2007-2008, 2009-2010, 2011-2012, 2013-2014, and 2015-2016 cycles. Out of the initial NHANES dataset, consisting of 79,648 participants, we excluded 39,749 subjects to focus our analysis exclusively on the adult population aged 20 years or older. Additionally, we further excluded participants without data on 2,4-DCP or 2,5-DCP (*n* = 29,341). Finally, 10,428 participants (5084 men and 5344 women) were included in the current study (Supplemental Fig. 1).

### Assessment of p-DCB

Urinary 2,5-DCP and 2,4 DCP were measured to evaluate the level of p-DCB exposure. Urine samples of each individual were collected and stored at − 20 °C for further study. The preparation, extraction, and measurement of urine samples were documented in the NHANES website [22]. In detail, the urine sample was treated using the on-line solid phase extraction (SPE) and the concentration of 2,5-DCP and 2,4 DCP were measured by HPLC linked tandem mass spectrometry [23]. The lower limit of detection (LLOD) for 2,5-DCP and 2,4 DCP was 0.2 ng/ml (for details, refer to https://wwwn.cdc.gov/Nchs/Nhanes/2015-2016/EPHPP_I.htm). The intra- and inter-assay coefficients of variation (CV) for 2,5-DCP and 2,4 DCP were in the range of 2.4%-3.4%. The urinary creatinine was measured using the Roche/Hitachi Modular P Chemistry Analyzer (Roche, Indianapolis, USA) and used to adjust the concentration of p-DCB exposure. The intra- and inter-assay CV for creatinine were in the range of 0.9%-3.0%. The urinary creatinine was measured using the Roche/Hitachi Modular P Chemistry Analyzer (Cobas 6000 analyzer, Roche, USA) and used to adjust the concentration of p-DCB exposure. The intra- and inter-assay CV for creatinine were in the range of 0.9%-3.0%.

### Ascertainment of outcome

Data on waist circumference (cm), fasting plasma glucose (FPG) (mg/dL), total cholesterol (TC), triglycerides (TG) (mg/dL), and high-density lipoprotein cholesterol (HDL-C; mg/dL) have been described on the NHANES website. In brief, TC, TG, and HDL-C were analyzed enzymatically in serum by spectrophotometric measurement of the colour of a reaction byproduct using Cholesterol Reagent (Part #467825), Trig/GB reagent (Roche product #1877771), HDL-C plus 3rd generation reagent kit (Roche product #04713214) on a Roche/Hitachi Modular P Chemistry Analyzer (Roche, Indianapolis, USA). Low-density lipoprotein cholesterol (LDL-C) levels were calculated from measured values of TC, TG and HDL-C based on the Friedwald equation ([LDL-C] = [TC] − [HDL-C] − [TG/5]) [24]. Glycohemoglobin (%) were analyzed with high-performance liquid chromatography 723G8 (Tosoh Bioscience, South San Francisco, CA.). Fasting glucose levels were measured using DxC 800 Chemistry Analyzer (Beckman Coulter, Indianapolis, USA). The intra- and inter- assay CVs were < 3.8% and < 2.2% for triglycerides; < 1.3% and < 1.5% for total cholesterol; < 4.6% and < 2.8% for HDL- C; 1.6% and < 1.3% for glycohemoglobin; and < 2.9% and < 1.1% for fasting glucose levels, respectively. The case of MetS was identified using the criteria of National Cholesterol Education Program Adult Treatment Panel III guidelines (NCEP/ATP III) [25]. Participants who matched three or more of the following five criteria were diagnosed as having MetS:

1. systolic blood pressure (SBP) ≥ 130 mmHg or diastolic blood pressure (DBP) ≥ 85 mmHg, or use of antihypertensive agents;
2. FPG ≥ 110 mg/dL, use of insulin or hypoglycemic drugs, or diagnosis of diabetes;
3. waist circumference ≥ 102 cm for males or 88 cm for females;
4. triglycerides ≥ 150 mg/dL;
5. HDL-C < 40 mg/dL for males or < 50 mg/dL for females.

### Covariates

Baseline information on age, sex, race, education, family poverty-income ratio (PIR), physical activity, smoking, alcohol drinking status, and medical history were collected by a household interview questionnaire (for details, refer to https://wwwn.cdc.gov/Nchs/Nhanes/2015-2016/Questionnaire Data). The smoking status was assessed using the concentration of serum cotinine (ng/mL) [26]. Participants self-reported cases of CVD or cancer. The total energy and fat intake were assessed using the average of two consecutive 24-h diet questionnaires.

### Statistical analysis

In this study, we addressed the intricate multistage probability sampling strategy of NHANES by incorporating the sampling weights, strata, and primary sampling units created by the National Center for Health Statistics (NCHS) into all our statistical analyses. Due to the skewness of the data, the urinary concentrations of 2,5-DCP and 2,4-DCP were transformed using the natural logarithm (ln). This transformation helps to normalize the distribution of the data and improve its statistical analysis. Total dichlorophenol was calculated using the sum of 2,5-DCP and 2,4 DCP. We conducted continuous (each 1-unit increase) and categorical (across quartiles) analyses to assess the odds ratios (ORs) and confidence intervals (CIs) of MetS prevalence related to p-DCB exposure using multivariate-adjusted logistic regression models. Known or suspected confounders were considered according to previous literature and biological plausibility. We used a Directed Acyclic Graph to show the hypothesized associations between p-DCB, confounders, and MetS prevalence (Supplemental Fig. 2). Finally, potential factors included urinary creatinine concentration, sex, age, race, education, PIR, physical activity, smoking, and drinking status, total energy intake and total fat intake. The first model was adjusted urinary creatinine concentration, model 2 was further adjusted sex, age, and race, model 3 was adjusted model 2 plus education, PIR, physical activity, smoking, and drinking status, and model 4 (full model) was adjusted for total energy intake and total fat intake based on model 3. Missing indicator categories were used for missing covariate data. We utilized restricted cubic spline models to investigate the dose-response of p-DCB exposure with MetS. We further tested associations between p-DCB exposure and MetS components. Moreover, Additionally, we examined the correlation between p-DCB exposure and MetS indicators, such as systolic blood pressure (SBP), FPG, triglycerides, waist circumference, glycohemoglobin, and HDL-C.

Furthermore, we also performed weighted quantile sum (WQS) regression and Quantile G-computation (qgcomp) to evaluate the combined effects of multiple p-DCB metabolites as the previous study described [19]. In brief, the sample size was randomly split into a training dataset (40%, *n* = 4171) and a validation dataset (60%, *n* = 6257). Bootstrapping was performed to evaluate the weights for 2,5-DCP and 2,4 DCP in the mixture using the training dataset. In current study, we implemented and evaluated both a positive and a negative WQS score [27]. For qgcomp analyses, the qgcomp.noboot function was used to evaluate exposure impacts between each investigated p-DCB metabolites and MetS. To illustrate the joint impact of 2,5-DCP and 2,4 DCP on MetS, a figure was generated utilizing g-computation and bootstrap variance with B iterations up to 10,000 [28]. In addition, subgroup and sensitivity analyses were conducted to test the robustness of the current result. Firstly, we investigated whether the associations changed stratified by age, sex, education, PIR, physical activity, smoking status, and drinking status. Subgroup analysis was used to assess whether the associations observed were consistent across different subgroups or if there were any subgroup-specific effects. The interaction was evaluated using the likelihood-ratio test. Additionally, sensitivity analyses were performed by accounting for CVD history, cancer history, hypoglycemic agents, and antihypertensive agents. In addition, pregnant individuals, those with extreme total energy intake, and individuals with extreme BMI were excluded to enhance the validity of our findings. Finally, we applied threshold regression to estimate the cutoff thresholds for the investigated p-DCB exposure levels relevant to MetS.

All statistical analyses were performed using SAS version 9.4 (SAS Institute Inc., Cary, NC). A two-sided p-value less than 0.05 was considered statistically significant. WQS and qgcomp analyses was conducted by R version 3.5.1 (The Comprehensive R Archive Network: http://cran.r-project.org) using gWQS and qgcomp package.

## Results

### Population characteristics

In the NHANES study, among the 10,428 participants, with an average of age 49.0 ± 17.8 years, 2,861 (27.1%) participants were diagnosed as MetS patients. Table 1 concluded the baseline characteristics of the current population classified by MetS status. Individuals with MetS were more often older and had higher levels of SBP, fasting glucose, waist circumference, and triglycerides compared with not MetS patients. They were less likely to be male, educated, physically active, and alcohol drinkers and had a lower income and total energy intake. Moreover, MetS participants had higher concentrations of 2,5-dichlorophenol, 2,4-dichlorophenol, and total dichlorophenol compared with the healthy population (mean: 247.1 μg/L versus 151.7 μg/L, 7.3 versus 4.5, and 254.5 versus 156.2, respectively).

**Table 1:** Population characteristics by metabolic syndrome status in NHANES 2003–2016 (*n* = 10,428)

**Characteristics**	**Overall (*n* = 10,428)**	**Non-MetS (*n* = 7,607)**	**MetS (*n* = 2,821)**	*P*-value	Adjusted *p*-values^a^
**Age (years)**	49.0 ± 17.8	46.0 ± 17.8	57.0 ± 15.4	< 0.001	0.005
**Male (%)**	48.8	49.4	47.0	0.026	0.078
**Education levels (%)**				0.008	0.032
Under high school	10.9	9.5	14.6		
High school	23.1	22.6	24.3		
Above high school	51.1	53.9	43.5		
**Race/ethnicity (%)**					
Mexican American	15.7	15.2	16.9		
Non-Hispanic white	42.6	42.3	43.2		
Non-Hispanic black	21.9	21.4	23.3		
Others	19.8	21.0	16.6		
**PIR**				< 0.001	0.005
< 1.52	43.7	42.3	47.4		
1.52 to 3.48	27.6	27.4	28.2		
> 3.48	28.7	30.3	24.4		
**Smoking Status (%)**				< 0.001	0.005
Never	29.2	28.4	31.5		
Former	46.7	47.4	44.9		
Active	24.0	24.2	23.6		
**Alcohol Drinking Status (%)**				< 0.001	0.005
Never	10.3	14.3	24.1		
Former	6.7	6.0	8.4		
Active	60.9	63.9	52.8		
**Physical activity (%)**				< 0.001	0.005
Never	34.4	31.2	43.1		
Moderate	30.9	30.1	33.4		
Vigorous	34.2	38.3	22.9		
**Cancer**				< 0.001	0.005
Yes	9.03	7.6	12.9		
No	90.97	92.4	87.1		
**Cardiovascular disease**				< 0.001	0.005
Yes	10.8	7.1	20.7		
No	89.2	92.9	79.3		
**Urinary creatinine (mg/dL)**	125.4 ± 80.0	125.9 ± 81.8	123.7 ± 75.0	0.199	0.199
**ln (Urinary creatinine)**	0.1 ± 0.1	0.1 ± 0.1	0.1 ± 0.1	0.199	0.199
**Indicator of Metabolic Syndrome**					
Systolic blood pressure (mmHg)	123.9 ± 18.5	120.9 ± 17.2	132.0 ± 19.2	< 0.001	0.005
Diastolic blood pressure (mmHg)	69.7 ± 13.1	69.2 ± 12.2	70.7 ± 15.1	< 0.001	0.005
Fasting glucose (mg/dL)	109.2 ± 36.2	99.8 ± 22.0	129.0 ± 49.8	< 0.001	0.005
Waist circumference (cm)	99.2 ± 16.4	94.7 ± 14.7	111.2 ± 14.5	< 0.001	0.005
Triglycerides (mg/dL)	127.8 ± 104.3	100.5 ± 69.5	185.5 ± 137.0	< 0.001	0.005
High density cholesterol (mg/dL)	53.1 ± 16.2	56.5 ± 16.2	43.7 ± 12.0	< 0.001	0.005
**Diet**					
Total energy intake, kcal/day	2113.8 ± 1017.0	2164.8 ± 1036.0	1978.2 ± 951.7	< 0.001	0.005
Total fat intake, g/day	79.8 ± 47.3	81.1 ± 48.1	76.3 ± 44.8	< 0.001	0.005
**Dichlorophenol biomarkers**					
2,5-dichlorophenol (μg/L)	177.5 ± 1215.7	151.7 ± 1125.4	247.1 ± 1429.1	< 0.001	0.005
2,4-dichlorophenol (μg/L)	5.3 ± 32.2	4.5 ± 27.8	7.3 ± 41.8	< 0.001	0.005
ln (2,5-dichlorophenol)	2.0 ± 2.2	1.9 ± 2.2	2.2 ± 2.3	< 0.001	0.005
ln (2,4-dichlorophenol)	-0.1 ± 1.5	-0.2 ± 1.4	0.0 ± 1.5	< 0.001	0.005
Total Dichlorophenol (μg/L)	182.8 ± 1246.8	156.2 ± 1152.1	254.5 ± 1469.9	< 0.001	0.005

Data are expressed as mean ± SD or numbers with percentages. *PIR* poverty-income ratio

^a^Adjusted *p*-values were assessed by Benjamini–Hochberg procedure

### Associations of p-DCB biomarkers with MetS and its components

**Fig. 1:**
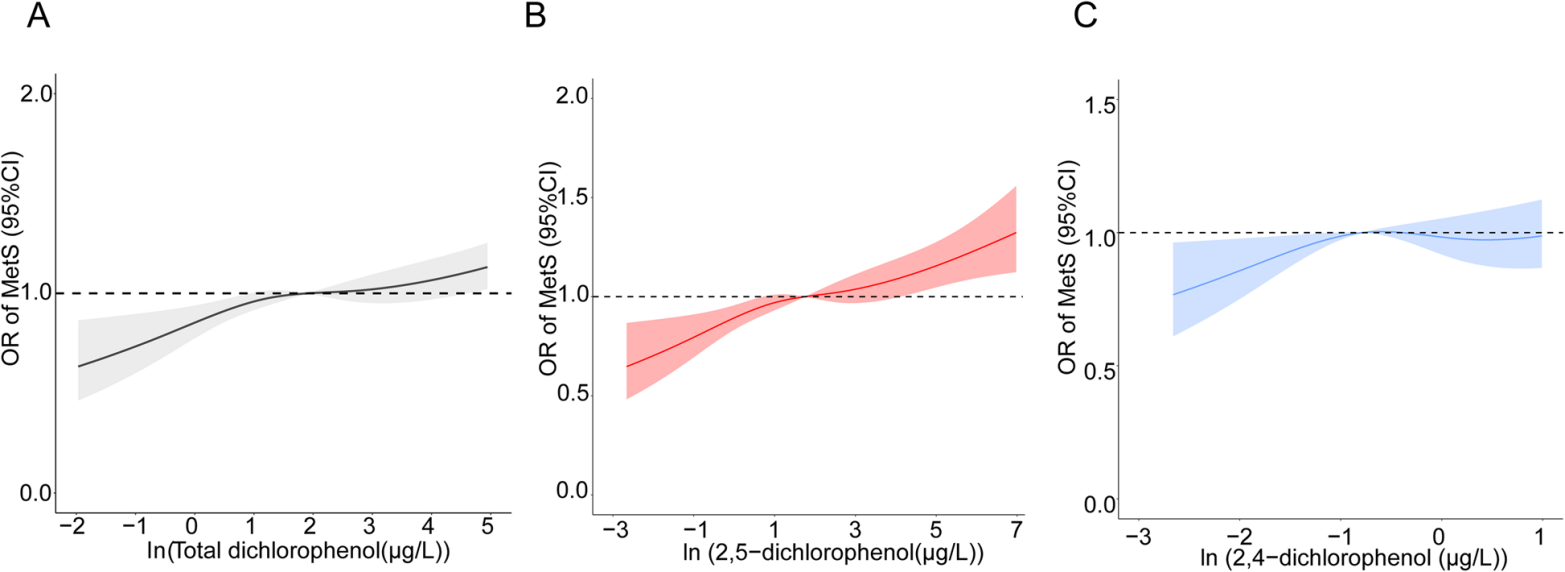
Associations between log-transformed Para-dichlorobenzene biomarkers and the prevalence of MetS. Odds ratios were estimated by restricted-cubic-spline regression after adjustment for creatinine concentration, age (years), gender (male or female), race (non-Hispanic Black, non-Hispanic White, Mexican American, or others), education (under high school, high school, or above high school), PIR (< 1.52, 1.52 to 3.48, or > 3.48), physical activity (never, moderate, or vigorous), smoking (non-smoker, former smoker, or active smoker), drinking (abstainer or active drinker) status, total energy intake and total fat intake. Shaded areas represent 95% confidence intervals. CI, confidence interval; PIR, poverty-income ratio, OR, odds ratio; MetS, metabolic syndrome

After adjusting the urinary creatinine, a 1-unit increase in 2,5-DCP and total dichlorophenol was related to a 5% higher MetS prevalence (Table 2). After adjustment for the lifestyle and dietary factors (model 4), the association between the total dichlorophenol and MetS prevalence is not significant, while the higher 2,5-DCP concentrations still had a higher prevalence of MetS. In the category analyses, we also observed significant and positive associations between the 2,5-DCP exposure and MetS prevalence. The multivariate-adjusted ORs (95% CIs) of MetS across increasing quartiles were 1.09 (0.93-1.28), 1.22 (1.00-1.49), and 1.34 (1.04-1.73) for 2,5-DCP in the full model (model 4, *P* = 0.018 for trend). Moreover, The restricted cubic spline (RCS) model, including p-DCB biomarkers as continuous variables, assessed the dose-response relation between p-DCB exposure and MetS prevalence and showed similar trends with the category analyses (Fig. 1). To investigate the potential mechanism of p-DCB inducing the MetS prevalence, we also conducted the analyses on the association between p-DCB biomarkers and MetS components (Table 3). Individuals with cases of elevated blood pressure (EBP), high fasting glucose (HFG), abdominal obesity, hypertriglyceridemia, and low HDL-C were 4,831 (46.3%), 1,663 (15.9%), 5,428 (15.9%), 1,194 (11.4%), and 3,569 (34.2%), respectively. The higher exposure of 2,5-DCP was positively associated with a higher prevalence of abdominal obesity (OR_Q4vsQ1_ = 1.23, 95% CI: 1.03-1.48, *P* = 0.017 for trend), whereas the levels of total dichlorophenol had a higher prevalence of HFG (OR_Q4vsQ1_ = 1.25, 95% CI: 0.96-1.62, *P* = 0.040 for trend).

**Table 2:** Multivariate-adjusted Odds ratios (95% CI) of associations between dichlorophenol biomarkers and the prevalence of MetS in NHANES 2003–2016 (*n* = 10,428)

**Biomarkers**^**a**^	Effect estimates by continuous metabolites	Effect estimates (95% CI) by quantiles of metabolites					
		Q1	Q2	Q3	Q4	*P*-trend	Adjust *P*-trend^b^
**2,5-dichlorophenol (μg/L)**		< 1.5	1.6–5.4	5.5–25.2	> 25.3		
** Case/N**	2,821/10,428	632/2,644	696/2,569	710/2610	783/2,605		
Model 1	1.05 (1.02–1.08)	1	1.10 (0.94–1.29)	1.21 (1.00–1.46)	1.26 (1.06–1.51)	0.009	0.027
Model 2	1.03 (1.00–1.06)	1	1.11 (0.95–1.30)	1.22 (1.01–1.47)	1.30 (1.09–1.56)	0.005	0.027
Model 3	1.02 (0.99–1.06)	1	1.08 (0.92–1.26)	1.16 (0.97–1.39)	1.21 (1.00–1.45)	0.039	0.078
Model 4	1.05 (1.00–1.10)	1	1.09 (0.93–1.28)	1.22 (1.00–1.49)	1.34 (1.04–1.73)	0.018	0.043
**2,4-dichlorophenol (μg/L)**		< 0.3	0.4–0.7	0.8–1.8	> 1.9		
** Case/N**	2,821/10,428	697/2,704	683/2,536	711/2,644	730/2,544		
Model 1	1.04 (0.99–1.08)	1	1.04 (0.88–1.23)	1.04 (0.86–1.25)	1.09 (0.90–1.31)	0.44	0.528
Model 2	0.99 (0.95–1.04)	1	1.04 (0.88–1.23)	1.04 (0.87–1.26)	1.09 (0.91–1.32)	0.387	0.516
Model 3	0.98 (0.94–1.03)	1	1.01 (0.85–1.21)	1.01 (0.84–1.21)	1.01 (0.83–1.21)	0.979	0.997
Model 4	0.98 (0.94–1.03)	1	0.96 (0.80–1.14)	0.91 (0.74–1.13)	0.83 (0.65–1.06)	0.997	0.997
**Total Dichlorophenol (μg/L)**		< 2.1	2.1–6.4	6.4–26.9	> 27		
** Case/N**	2,821/10,428	639/2,637	686/2,560	713/2,623	783/2,608		
Model 1	1.05 (1.02–1.08)	1	1.06 (0.90–1.25)	1.19 (1.00–1.41)	1.25 (1.05–1.49)	0.008	0.027
Model 2	1.03 (0.99–1.06)	1	1.07 (0.91–1.26)	1.20 (1.01–1.42)	1.28 (1.07–1.54)	0.005	0.027
Model 3	1.02 (0.98–1.05)	1	1.03 (0.88–1.22)	1.14 (0.96–1.34)	1.18 (0.99–1.42)	0.046	0.079
Model 4	1.02 (0.98–1.05)	1	1.02 (0.86–1.21)	1.13 (0.95–1.33)	1.17 (0.98–1.41)	0.053	0.080

*CI* confidence interval, *OR* Odd ratio, *Q* quintile, *PIR* poverty-income ratio

Model 1 was adjusted urinary creatinine concentration

Model 2 was adjusted for covariates in Model l plus age (years), gender (male or female) and race (non-Hispanic Black, non-Hispanic White, Mexican American, or others)

Model 3 was adjusted for covariates in Model 2 plus education (under high school, high school, or above high school), PIR (< 1.52, 1.52 to 3.48, or > 3.48), physical activity (never, moderate, or vigorous), smoking (non-smoker, former smoker, or active smoker), and drinking (abstainer or active drinker) status

Model 4 was adjusted for covariates in Model 3 plus total energy intake and total fat intake

^a^Biomarkers were log-transformed and individually included in the multivariable logistic regression model

^b^Adjusted *p*-values were assessed by Benjamini–Hochberg procedure

**Table 3:** Multivariate-adjusted odds ratios (95% CI) for associations between dichlorophenol biomarkers and individual components of MetS prevalence in NHANES 2003–2016

^a^Biomarkers	Elevated blood pressure (*n* = 4,831)			High fasting glucose (*n* = 1,663)			Abdominal obesity (*n* = 5,428)			Hypertriglyceridemia (*n* = 1,194)			Low HDL-C (*n* = 3,569)		
	OR (95% CI)	*p*_trend_	Adjust *p*_trend_^b^	OR (95% CI)	*p*_trend_	Adjust *p*_trend_^b^	OR (95% CI)	*p*_trend_	Adjust *p*_trend_^b^	OR (95% CI)	*p*_trend_	Adjust *p*_trend_^b^	OR (95% CI)	*p*_trend_	Adjust *p*_trend_^b^
2,5-dichlorophenol (μg/L)		0.862	0.862		0.202	0.38		0.017	0.085		0.378	0.472		0.228	0.38
Q1	1			1			1			1			1		
Q2	0.97 (0.82–1.14)			0.97 (0.81–1.17)			1.13 (0.97–1.31)			1.16 (0.94–1.44)			0.98 (0.83–1.15)		
Q3	1.08 (0.91–1.28)			1.12 (0.89–1.40)			1.20 (1.02–1.40)			1.23 (0.99–1.52)			1.11 (0.95–1.30)		
Q4	0.98 (0.80–1.19)			1.13 (0.89–1.44)			1.23 (1.03–1.48)			1.06 (0.86–1.29)			1.07 (0.89–1.29)		
2,4-dichlorophenol (μg/L)		0.479	0.479		0.159	0.392		0.318	0.398		0.235	0.392		0.056	0.28
Q1	1			1			1			1			1		
Q2	1.02 (0.85–1.22)			1.09 (0.85–1.40)			0.97 (0.83–1.14)			1.05 (0.83–1.33)			1.00 (0.86–1.16)		
Q3	1.05 (0.87–1.26)			1.15 (0.85–1.55)			0.99 (0.84–1.17)			0.93 (0.72–1.19)			0.94 (0.81–1.10)		
Q4	0.91 (0.74–1.11)			1.26 (0.92–1.72)			0.90 (0.75–1.07)			0.88 (0.68–1.15)			0.86 (0.72–1.01)		
Total dichlorophenol (μg/L)		0.143	0.238		0.04	0.155		0.062	0.155		0.58	0.58		0.419	0.524
Q1	1			1			1			1			1		
Q2	1.03 (0.89–1.20)			1.02 (0.83–1.27)			0.95 (0.83–1.09)			1.25 (0.98–1.59)			0.95 (0.81–1.11)		
Q3	1.12 (0.96–1.30)			1.25 (0.99–1.59)			1.10 (0.93–1.29)			1.15 (0.92–1.43)			1.09 (0.93–1.28)		
Q4	1.13 (0.94–1.35)			1.25 (0.96–1.62)			1.17 (0.97–1.40)			1.07 (0.88–1.31)			1.03 (0.85–1.25)		

*CI* confidence interval, *OR* Odd ratio, *Q* quintile, *PIR* poverty-income ratio

Adjusted covariates: urinary creatinine concentration, age (years), gender (male or female) and race (non-Hispanic Black, non-Hispanic White, Mexican American, or others), education (under high school, high school, or above high school), PIR (< 1.52, 1.52 to 3.48, or > 3.48), physical activity (never, moderate, or vigorous), smoking (non-smoker, former smoker, or active smoker), and drinking (abstainer or active drinker) status, total energy intake, and total fat intake

^a^Biomarkers were log-transformed and individually included in the multivariable logistic regression model

^b^Adjusted *p*-values were assessed by Benjamini-Hochberg procedure

### Association between p-DCB biomarkers with MetS in WQS and qgcomp analyses

Similar to the results of p-DCB biomarkers as continuous variable in RCS model (Fig. 1), the mixed effects of 2,5-DCP and 2,4-DCP were positively associated with MetS prevalence in both the WQS and gqcomp models. In fully adjusted models, the ORs (95% CIs) of MetS and mixed effects p-DCB metabolites were 1.02 (1.00-1.03) for WQS (positive weight) and 1.02 (1.01-1.02) for gqcomp model (Table S1). Both WQS and gqcomp model showed the 2,5-DCP received the highest positive weights (Fig. S3).

### Association between p-DCB biomarkers and MetS indicators

We further conducted the analyses to test the relation between p-DCB Biomarkers and MetS factors (Table S2). The 2,5-DCP concentration was associated with higher SBP (*r* = 0.022, *P* = 0.027), waist circumference (*r* = 0.099, *P* < 0.001), glycohemoglobin (*r* = 0.027, *P* = 0.008), and lower HDL-C (*r* = -0.059, *P* < 0.001) after adjusting for the full covariates. The associations of 2,4 DCP with MetS indicators including waist circumference (*r* = 0.080, *P* < 0.001) and lower HDL-C (*r* = -0.039, *P* < 0.001) showed a similar trend. Besides, positive correlations were found between total dichlorophenol and waist circumference (*r* = 0.094, *P* < 0.001) and glycohemoglobin (*r* = 0.023, *P* = 0.021) while a negative association for HDL-C (*r* = -0.053, *P* < 0.001).

### Subgroup analyses and sensitivity analyses

**Fig. 2:**
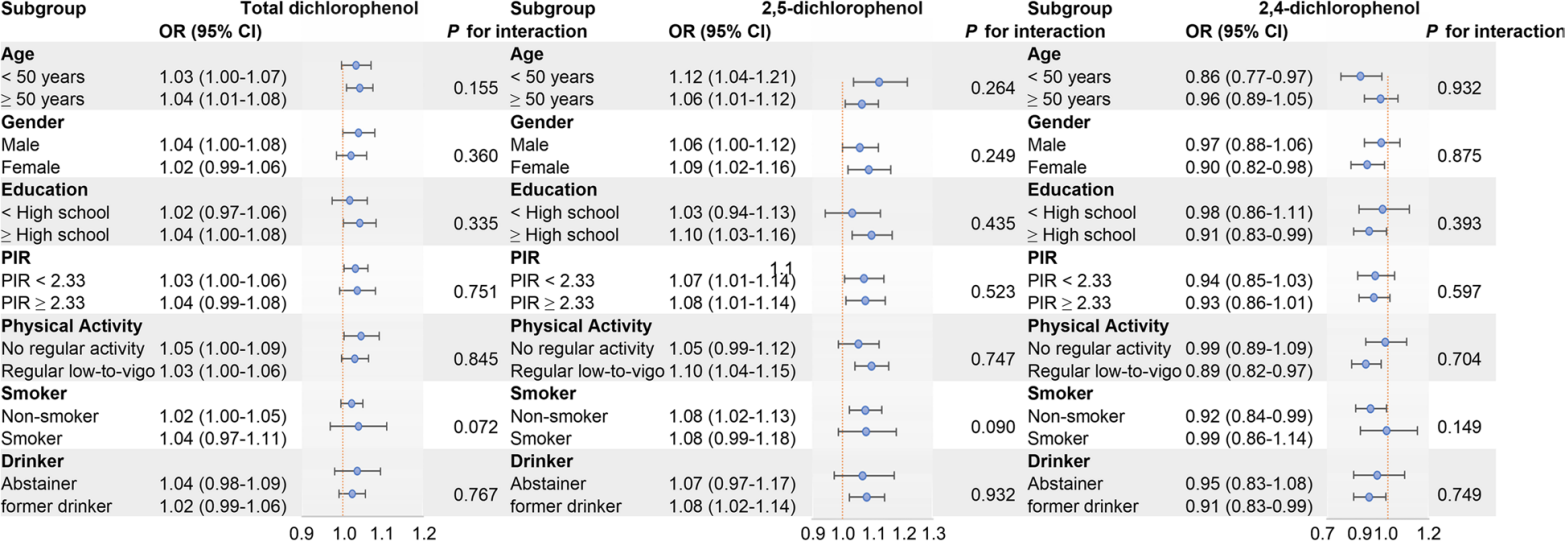
Subgroup analyses for the associations between the Para-dichlorobenzene and the prevalence of MetS in NHANES 2003-2016. Adjusted covariates: creatinine concentration, age (years), gender (male or female), race (non-Hispanic Black, non-Hispanic White, Mexican American, or others), education (under high school, high school, or above high school), PIR (< 1.52, 1.52 to 3.48, or > 3.48), physical activity (never, moderate, or vigorous), smoking (non-smoker, former smoker, or active smoker), drinking (abstainer or active drinker) status, total energy intake and total fat intake. Shaded areas represent 95% confidence intervals. CI, confidence interval; PIR, poverty-income ratio, OR, odds ratio; MetS, metabolic syndrome

In the subgroup analyses, we observed the positive associations between 2,5-DCP concentration were similarly stratified by age, sex, education, income, exercise, smoking or drinking status (all *P* for interaction > 0.05) (Fig. 2). In the sensitivity analyses, the observed associations were not substantially influenced by the additional adjustment for CVD and cancer or the use of glucose lowering drug and antihypertensive agents (Table S3). The consistent results after excluding individuals with extreme energy intake, BMI, or pregnancy supported the validity of the positive associations between 2,5-DCP exposure and MetS prevalence (Table S4).

## Discussion

This study examined the relations between p-DCB Biomarkers and the prevalence of MetS and its indicators. Using the data of 10,428 participants with 2,861 cases of MetS, we observed that higher 2,5-DCP levels were positively associated with MetS prevalence. After adjusting demographic, lifestyle, and dietary confounders, individuals in the highest versus lowest quartiles of 2,5-DCP concentrations had a 34% higher prevalence of MetS. Moreover, the 2,5-DCP exposure was associated with higher abdominal obesity prevalence and the increase of MetS factors including systolic blood pressure, waist circumference, and glycohemoglobin.

Recently, numerous epidemiological studies linking p-DCB exposure to chronic health burdens have attracted global concern [8, 12, 20]. A previous cross-sectional NHANES study conducted by Wei et al. (2016) reported that higher 2,5-DCP concentration showed a significant association with diabetes prevalence (OR: 1.59, 95% CI: 1.06-2.40) [12]. Our findings also found that total dichlorophenol was related to a 25% higher prevalence of diabetes (Table 3). The positive correlation between the total dichlorophenol, especially 2,5-DCP and glycohemoglobin may explain the adverse effects of dichlorophenol for the risk of developing diabetes [29]. Another work based on the NHANES study (2007–2010) found that 2,5-DCP had an 84% (95% CI: 26%-170%) higher prevalence of CVD after adjusting potential confounders [30]. Consistently, we found that higher urinary 2,5-DCP levels are positively associated with systolic blood pressure. Additionally, epidemiological evidence revealed a significant and positive association between p-DCB exposure and obesity risk. A previous cross-sectional study collected the data from NHANES (2005–2008) to investigate the association between dichlorophenol pesticides and the prevalence of obesity [20]. Consistent with our results, Wei et al. demonstrated that p-DCB metabolite 2,5-DCP not 2,4-DCP was positively associated with obesity prevalence [20]. A recent study based on Korean girls also reported that chlorophenol exposure had a higher risk of obesity by affecting waist circumference [31]. In the current study, we also revealed the significant and positive relation between the 2,5-DCP and 2,4-DCP and waist circumference. Overall, these epidemiological studies suggested that p-DCB exposure may pose a potential risk for the development of metabolic disorders. Currently, there is limited research examining the impact of p-DCB on MetS, and there are no defined cutoff criteria for clinically relevant exposure levels. In this study, the geometric mean of urinary concentrations of 2,5-DCP (5.5 μg/L) in U.S. adult was 1.2-fold higher than that in the German population based on the 1998 German Environmental Survey [17]. Previous studies suggested the thresholds of 2,5-DCP were 29.9 μg/L for diabetes, 13.4 μg/L for CVD, and 157.4 μg/L for cancer [12, 30]. However, our present study predicts that the p-DCB reference level must be lower than previously advised levels to avoid MetS prevalence (Table S5).

The metabolism of para-dichlorobenzene (p-DCB) in humans and animals involves oxidation, reduction, and conjugation reactions that result in the formation of several metabolites [32]. The primary metabolites of p-DCB include 2,5-dichlorophenol (2,5-DCP), 2,4-dichlorophenol (2,4-DCP), and 4-chlorophenol (4-CP) [33]. These metabolites are formed through the oxidative dechlorination of p-DCB by cytochrome P450 enzymes in the liver, followed by conjugation with glucuronic acid or sulfate in the liver and kidneys [32]. 2,5-DCP is the major metabolite of p-DCB and is excreted in the urine, accounting for approximately 90% of the dose in humans [17]. Other minor metabolites of p-DCB include 2,6-dichlorophenol, 3,5-dichlorocatechol, and 3-chlorocatechol, which are formed through further oxidation and cleavage reactions [33]. Previous studies showed that skin contact with 2,5-dichlorophenol can cause irritation and inflammation, and prolonged exposure can result in skin sensitization [34]. Moreover, 2,5-dichlorophenol may be associated with adverse effects on the endocrine system, which regulates hormone production, leading to reproductive and developmental problems, thyroid dysfunction, and other health issues [8, 30, 35]. In the current study, we also found that 2,5-DCP exposure was associated with dysglycolipidosis, thus leading to the MetS prevalence. On the other hand, 2,4-DCP has been shown to induce oxidative stress and inflammatory responses [36, 37]. Exposure to 2,4-DCP has been found to increase intracellular oxidative stress substances such as superoxide dismutase, catalase, and glutathione peroxidase [38]. These increased substances can lead to oxidative stress reactions and damage to cell components such as membranes, proteins, and DNA [39]. Our results of non-significant association of 2,4-DCP with MetS prevalence may be attributed to too low concentration of 2,4-DCP (5.3 ug/L). Overall, 2,5-DCP and 2,4-DCP can serve as biomarkers of p-DCB, used to assess the level of exposure, and the documented positive relationship of p-DCB and MetS prevalence could be mainly explained by the toxic effects of 2,5-DCP.

The positive association between 2,5-DCP and MetS prevalence in this study can be linked to the pathophysiology of dysglycolipidosis. Evidence has revealed that p-DCB affected thyroid gland functions and was negatively associated with free thyroxine [40], leading to a higher risk of MetS [41]. Previous studies also found that 2,5-DCP may lead to metabolic risk by the disturbance of glycolipid homeostasis consistent with the current study that 2,5-DCP concentrates were associated with the higher glycohemoglobin level [12] and lower HDL-C [7]. Dyslipidemia, such as TG/HDL-C, has become an important marker for the development of MetS risk [42]. The latest meta-analyses also concluded that pesticide exposure increased the risk by altering the HDL-C levels [43, 44]. p-DCB can be considered an obesogen because it has the potential to interfere with the body's natural processes for fat cell formation (adipogenesis) and the regulation of energy balance. This toxicant exposure may lead to alter activity of a group of nuclear hormone receptors known as peroxisome proliferator-activated receptors (PPARs) that play role in regulation of adipogenesis, and control of lipids and glucose metabolism [45]. In addition, p-DCB with EDCs activity potentially lead to deregulate pancreatic islet beta-cell function, development of peripheral Insulin resistance (IR), insulin production, beta-cell mass (compensatory hyperplasia/hypertrophy of beta cells) and impaired insulin output, insulin signaling, and increasing cell apoptosis [45, 46]. Insulin resistance can disrupt the balance of glucose metabolism and result in chronic hyperglycemia, which leads to oxidative stress [47] and causes an inflammatory response [48] that contributes to cellular damage [49]. Moreover, insulin resistance can also alter systemic lipid metabolism and thus causing the MetS [50]. Further cohort or case-control research is warranted to investigate the potential mechanisms of p-DCB exposure associated with higher MetS prevalence.

Our study has several strengths. We provided the largest and most extensive evaluation (*n* = 10,428) on the associations of the 2,5-DCP ratio, a urinary biomarker of exposure to p-DCB, with MetS and its components. Meanwhile, the high correlation between p-DCB biomarker and glycolipid indicators, such as waist circumference, glycohemoglobin, and HDL-C suggests the potential causality of the relation between p-DCB exposure and MetS prevalence. Moreover, excluding participants without the value of p-DCB biomarkers (including 2,5-DCP and 2,4-DCP) and MetS indexes (such as TC, TG, HDL-C, and FPG) could effectively assess relations between p-DCB and MetS prevalence. Finally, comprehensive information of covariates, including demographic, lifestyle, and dietary factors, used in the current study can allow us to investigate the realistic associations between p-DCB and MetS. In addition, some limitations are worth discussing. First, in NHANES, one time point urine sample was used to determine the concentration of 2,5-DCP and 2,4-DCP. Although we have adjusted the urinary creatinine in the model for better evaluation of p-DCB exposure [51], it might not represent long-term exposure to p-DCB. Thus, the repeat measurement of p-DCB exposure biomarkers or measurement of biomarkers in the blood is warranted to confirm the current results. Second, although we have strictly controlled for lifestyle and dietary factors in the multivariate-adjusted model, residual confoundings such as measurement and self-report errors were inevitable. Third, the generalizability of our findings was restricted to American descent. Fourth due to observational nature, the causality of the association between p-DCB exposure and MetS remains unclear. Finally, since the genes associated with p-DCBs are presently inaccessible, the molecular mechanisms linking these chemicals to MetS remain unclear, including the involvement of genes, miRNAs, and pathways. Thus, prospective studies and animal experiments need to elucidate the potential mechanism in the future.

## Conclusions

In this study, p-DCB exposure biomarkers, 2,5-DCP, were significantly positively associated with a higher prevalence of MetS among U.S. adults. Notably, highly positive correlation between 2,5-DCP and lower HDL-C and higher glycohemoglobin suggested the potential mechanism of p-DCB exposure induced glycolipid metabolism and cause the developing MetS. Further long-time follow up studies are warranted to verify our results and investigate potential mechanisms.

### Supplementary Information

**Additional file 1:** **Supplemental Figure 1. **Flow chart of study population. **Supplemental Figure 2. **Directed Acyclic Graphs for the Causal Effect of dichlorophenol with MetS prevalence. **Supplemental Figure 3.** WQS model regression positive weights (A) and negative weights (B) for p-DCB biomarkers and qgcomp model regression index weights (C). **Supplemental Table 1. **Associations of p-DCB biomarkers with MetS. **Supplemental Table 2. **Coefficients of dichlorophenol biomarkers for metabolic syndrome indicators from Spearman's rank correlation coefficient. **Supplemental Table 3. **Multivariate-adjusted ORs (95% CIs) for associations between dichlorophenol biomarkers and metabolic syndrome prevalence in sensitivity analyses. **Supplemental Table 4. **Multivariate-adjusted ORs (95% CIs) for associations between dichlorophenol biomarkers and metabolic syndrome prevalence in sensitivity analyses. **Supplemental Table 5. **Estimated cutoff thresholds for the investigated p-DCB that are relevant to MetS.

## Data Availability

All data are open access and available for download at url: https://www.cdc.gov/nchs/nhanes/index.htm (accessed on 18 June 2023).
