# Supplementary material for: Associations of dichlorophenol with metabolic syndrome based on multivariate-adjusted logistic regression: a U.S. nationwide population-based study 2003-2016
Source: Environ Health. 2023 Dec 15;22:88. doi: 10.1186/s12940-023-01037-z (PMC10722741; doi:10.1186/s12940-023-01037-z)
Supplement: Supplementary file 1 — Additional file 1: Supplemental Figure 1. Flow chart of study population. Supplemental Figure 2. Directed Acyclic Graphs for the Causal Effect of dichlorophenol with MetS prevalence. Supplemental Figure 3. WQS model regression positive weights (A) and negative weights (B) for p-DCB biomarkers and qgcomp model regression index weights (C). Supplemental Table 1. Associations of p-DCB biomarkers with MetS. Supplemental Table 2. Coefficients of dichlorophenol biomarkers for metabolic syndrome indicators from Spearman's rank correlation coefficient. Supplemental Table 3. Multivariate-adjusted ORs (95% CIs) for associations between dichlorophenol biomarkers and metabolic syndrome prevalence in sensitivity analyses. Supplemental Table 4. Multivariate-adjusted ORs (95% CIs) for associations between dichlorophenol biomarkers and metabolic syndrome prevalence in sensitivity analyses. Supplemental Table 5. Estimated cutoff thresholds for the investigated p-DCB that are relevant to MetS. [file 12940_2023_1037_MOESM1_ESM.docx]

**Supplemental Material**

**Associations of** **dichlorophenol with metabolic syndrome based on multivariate-adjusted logistic regression: A U.S. nationwide population-based study 2003-2016**


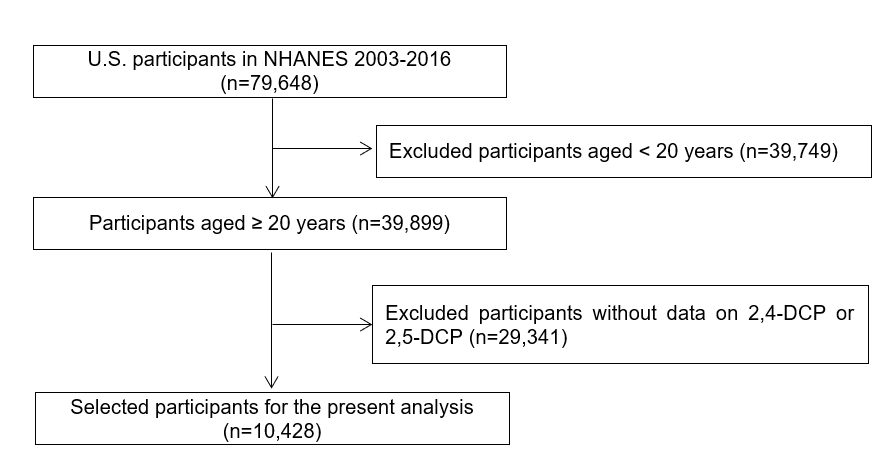


**Supplemental figure 1.** Flow chart of study population


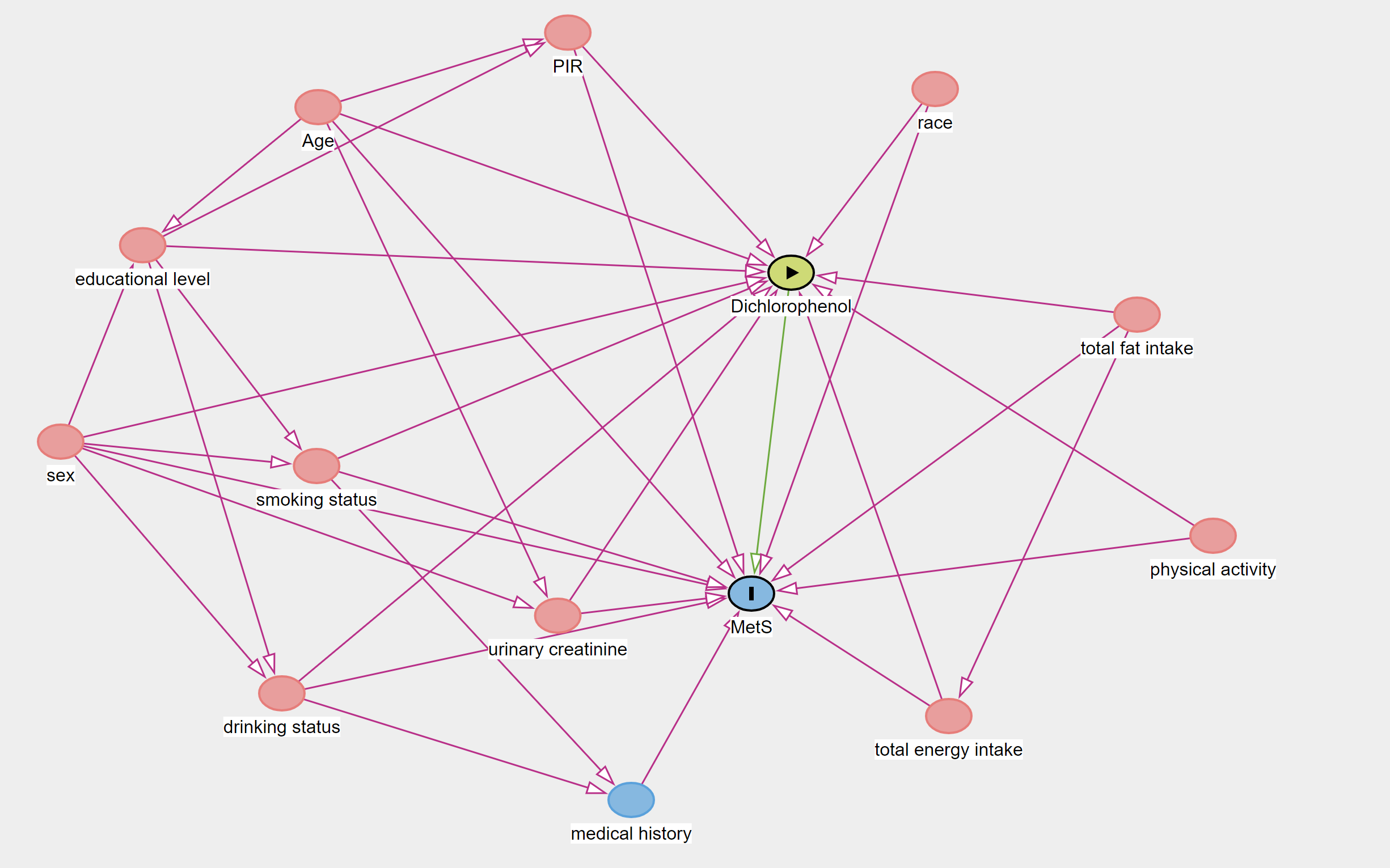


**Supplemental figure 2.** Directed Acyclic Graphs for the Causal Effect of dichlorophenol with MetS prevalence


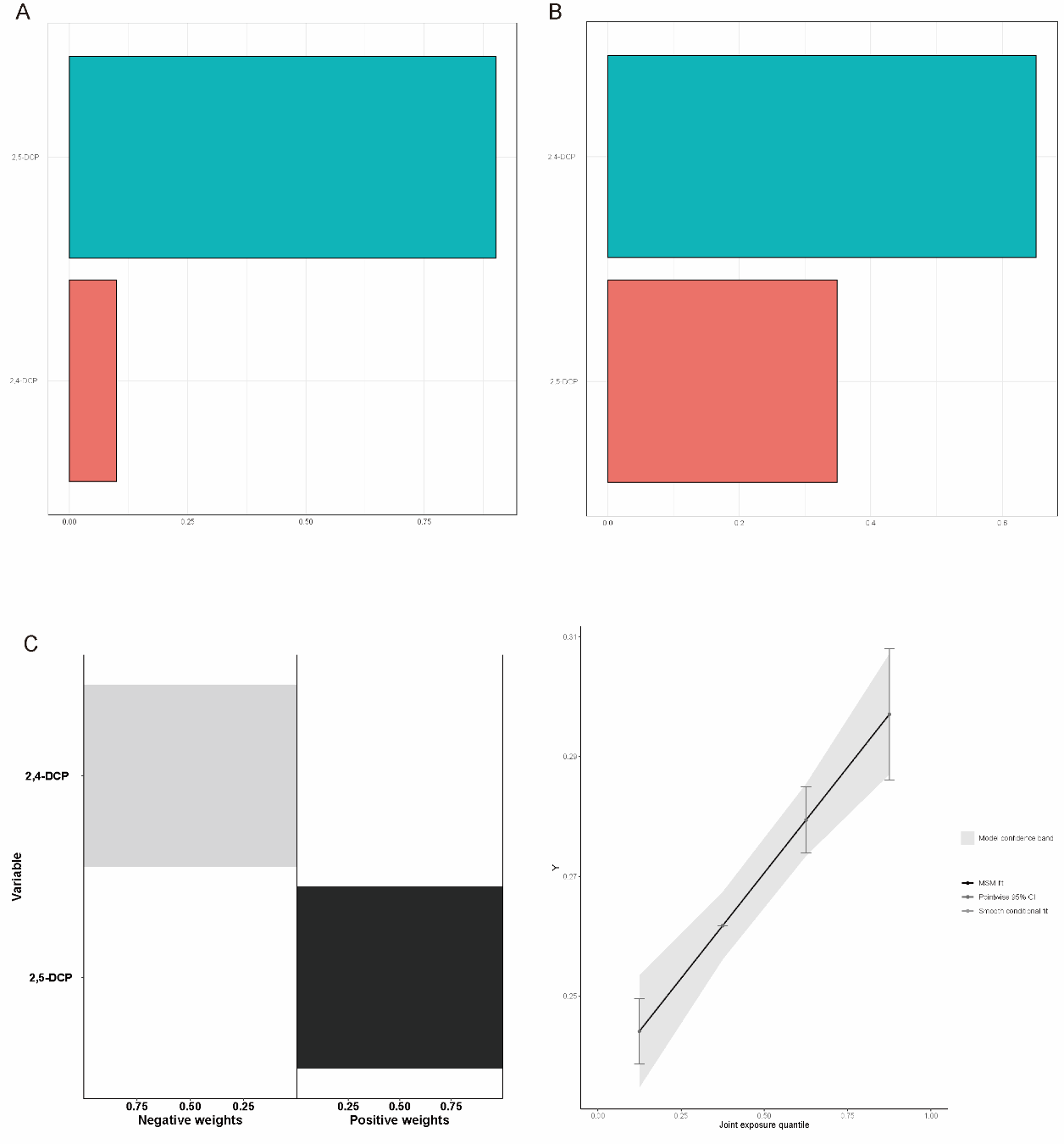


**Supplemental figure 3.** WQS model regression positive weights (A) and negative weights (B) for p-DCB biomarkers and qgcomp model regression index weights (C). Adjusted covariates: creatinine concentration, age (years), gender (male or female), race (non-Hispanic Black, non-Hispanic White, Mexican American, or others), education (under high school, high school, or above high school), PIR (< 1.52, 1.52 to 3.48, or > 3.48), physical activity (never, moderate, or vigorous), smoking (non-smoker, former smoker, or active smoker), drinking (abstainer or active drinker) status, total energy intake and total fat intake. CI, confidence interval; PIR, poverty-income ratio, OR, odds ratio; MetS, metabolic syndrome.

**Supplemental Table 2.** Associations of p-DCB biomarkers with MetS

| **Models** | **MetS** |
| --- | --- |
|  | **OR (95%CI)** |
| WQS positive weight | 1.02 (1.00-1.03) |
| WQS negative weight | 1.02 (1.00-1.03) |
| gqcomp | 1.02 (1.01-1.02) |

Adjusted covariates: creatinine concentration, age (years), gender (male or female), race (non-Hispanic Black, non-Hispanic White, Mexican American, or others), education (under high school, high school, or above high school), PIR (< 1.52, 1.52 to 3.48, or > 3.48), physical activity (never, moderate, or vigorous), smoking (non-smoker, former smoker, or active smoker), drinking (abstainer or active drinker) status, total energy intake and total fat intake. CI, confidence interval; PIR, poverty-income ratio, OR, odds ratio; MetS, metabolic syndrome.

**Supplemental Table 2.** Coefficients of dichlorophenol biomarkers for metabolic syndrome indicators from Spearman's rank correlation coefficient

| **Blood indicator** | **2,5-dichlorophenol (µg/L)** | | **2,4-dichlorophenol (µg/L)** | | **Total Dichlorophenol (µg/L)** | |
| --- | --- | --- | --- | --- | --- | --- |
|  | **r** | ***P*-value** | **r** | ***P*-value** | **r** | ***P*-value** |
| Systolic blood pressure (mmHg) | 0.022 | 0.027 | -0.001 | 0.960 | 0.004 | 0.698 |
| Diastolic blood pressure (mmHg) | -0.011 | 0.258 | -0.012 | 0.246 | -0.012 | 0.231 |
| Fasting glucose (mg/dL) | 0.016 | 0.259 | -0.001 | 0.921 | 0.012 | 0.388 |
| Waist circumference (cm) | 0.099 | < 0.001 | 0.080 | < 0.001 | 0.094 | < 0.001 |
| Triglycerides (mg/dL) | 0.027 | 0.062 | 0.019 | 0.199 | 0.025 | 0.089 |
| High density cholesterol (mg/dL) | -0.059 | < 0.001 | -0.039 | < 0.001 | -0.053 | < 0.001 |
| Total Cholesterol (mg/dL) | 0.013 | 0.365 | -0.017 | 0.091 | -0.011 | 0.263 |
| Low density cholesterol (mg/dL) | -0.012 | 0.233 | 0.001 | 0.970 | 0.013 | 0.375 |
| HbA1c (%) | 0.027 | 0.008 | 0.004 | 0.698 | 0.023 | 0.021 |

Adjusted covariates: creatinine concentration, age (years), gender (male or female), race (non-Hispanic Black, non-Hispanic White, Mexican American, or others), education (under high school, high school, or above high school), PIR (< 1.52, 1.52 to 3.48, or > 3.48), physical activity (never, moderate, or vigorous), smoking (non-smoker, former smoker, or active smoker), drinking (abstainer or active drinker) status, total energy intake and total fat intake. CI, confidence interval; PIR, poverty-income ratio, OR, odds ratio; MetS, metabolic syndrome.

**Supplemental Table 3. Multivariate-adjusted ORs (95% CIs) for associations between dichlorophenol biomarkers and metabolic syndrome prevalence in sensitivity analyses**

| **Biomarkers** | **Further adjustment for family history of CVD** | | **Further adjustment for family history of cancer** | | **Further adjustment for lower sugar drug** | | **Further adjustment for antihypertensive agents** | |
| --- | --- | --- | --- | --- | --- | --- | --- | --- |
|  | **OR (95%CI)** | ***p* _trend_** | **OR (95%CI)** | ***p* _trend_** | **OR (95%CI)** | ***p* _trend_** | **OR (95%CI)** | ***p* _trend_** |
| 2,5-dichlorophenol (µg/L) | | 0.029 |  | 0.028 |  | 0.002 |  | 0.027 |
| Q1 | 1 |  | 1 |  | 1 |  | 1 |  |
| Q2 | 1.05 (0.89-1.24) |  | 1.04 (0.88-1.23) |  | 1.14 (0.95-1.37) |  | 1.07 (0.90-1.27) |  |
| Q3 | 1.21 (0.98-1.50) |  | 1.21 (0.98-1.50) |  | 1.34 (1.08-1.66) |  | 1.25 (1.00-1.56) |  |
| Q4 | 1.31 (1.02-1.69) |  | 1.32 (1.02-1.70) |  | 1.48 (1.13-1.94) |  | 1.31 (1.01-1.70) |  |
| 2,4-dichlorophenol (µg/L) | | 0.057 |  | 0.043 |  | 0.076 |  | 0.047 |
| Q1 | 1 |  | 1 |  | 1 |  | 1 |  |
| Q2 | 0.91 (0.76-1.09) |  | 0.91 (0.75-1.09) |  | 0.88 (0.72-1.07) |  | 1.10 (1.01-1.21) |  |
| Q3 | 0.89 (0.72-1.10) |  | 0.87 (0.71-1.08) |  | 0.88 (0.71-1.09) |  | 0.89 (0.74-1.08) |  |
| Q4 | 0.76 (0.58-0.98) |  | 0.75 (0.58-0.97) |  | 0.76 (0.58-1.00) |  | 0.85 (0.69-1.04) |  |
| Total Dichlorophenol (µg/L) | | 0.227 |  | 0.241 |  | 0.009 |  | 0.185 |
| Q1 | 1 |  | 1 |  | 1 |  | 1 |  |
| Q2 | 0.98 (0.82-1.17) |  | 0.97 (0.81-1.15) |  | 1.06 (0.88-1.28) |  | 0.97 (0.80-1.18) |  |
| Q3 | 1.10 (0.92-1.30) |  | 1.09 (0.92-1.29) |  | 1.22 (1.02-1.46) |  | 1.12 (0.93-1.35) |  |
| Q4 | 1.09 (0.91-1.30) |  | 1.08 (0.90-1.30) |  | 1.25 (1.03-1.51) |  | 1.10 (0.90-1.33) |  |

Adjusted covariates: creatinine concentration, age (years), gender (male or female), race (non-Hispanic Black, non-Hispanic White, Mexican American, or others), education (under high school, high school, or above high school), PIR (< 1.52, 1.52 to 3.48, or > 3.48), physical activity (never, moderate, or vigorous), smoking (non-smoker, former smoker, or active smoker), drinking (abstainer or active drinker) status, total energy intake and total fat intake. CI, confidence interval; PIR, poverty-income ratio, OR, odds ratio; MetS, metabolic syndrome.

**Supplemental Table 4. Multivariate-adjusted ORs (95% CIs) for associations between dichlorophenol biomarkers and metabolic syndrome prevalence in sensitivity analyses**

| **Biomarkers** | **Exclusion of extreme energy intake (N= 9,093)** | | **Exclusion of extreme BMI (N=** **9,437)** | | **Exclusion of the pregnant (N=10,208)** | |
| --- | --- | --- | --- | --- | --- | --- |
|  | **OR (95%CI)** | ***p* _trend_** | **OR (95%CI)** | ***p* _trend_** | **OR (95%CI)** | ***p* _trend_** |
| 2,5-dichlorophenol (µg/L) |  | 0.012 |  | 0.034 |  | 0.030 |
| Q1 | 1.00 |  | 1.00 |  | 1.00 |  |
| Q2 | 1.01 (0.84-1.22) |  | 1.06 (0.87-1.28) |  | 1.04 (0.88-1.23) | |
| Q3 | 1.20 (0.96-1.50) |  | 1.21 (0.96-1.52) |  | 1.22 (0.99-1.51) | |
| Q4 | 1.42 (1.10-1.83) |  | 1.33 (1.02-1.75) |  | 1.31 (1.01-1.69) | |
| 2,4-dichlorophenol (µg/L) |  | 0.013 |  | 0.022 |  | 0.047 |
| Q1 | 1.00 |  | 1.00 |  | 1.00 |  |
| Q2 | 0.91 (0.75-1.10) |  | 0.86 (0.70-1.05) |  | 0.90 (0.75-1.09) | |
| Q3 | 0.88 (0.71-1.10) |  | 0.84 (0.67-1.05) |  | 0.88 (0.71-1.09) | |
| Q4 | 0.68 (0.53-0.88) |  | 0.72 (0.55-0.93) |  | 0.74 (0.57-0.97) | |
| Total Dichlorophenol (µg/L) |  | 0.251 |  | 0.418 |  | 0.275 |
| Q1 | 1.00 |  | 1.00 |  | 1.00 |  |
| Q2 | 0.96 (0.79-1.16) |  | 0.98 (0.81-1.19) |  | 0.98 (0.82-1.17) | |
| Q3 | 1.06 (0.87-1.29) |  | 1.04 (0.87-1.23) |  | 1.10 (0.92-1.31) | |
| Q4 | 1.10 (0.90-1.34) |  | 1.07 (0.88-1.29) |  | 1.07 (0.89-1.29) | |

Adjusted covariates: creatinine concentration, age (years), gender (male or female), race (non-Hispanic Black, non-Hispanic White, Mexican American, or others), education (under high school, high school, or above high school), PIR (< 1.52, 1.52 to 3.48, or > 3.48), physical activity (never, moderate, or vigorous), smoking (non-smoker, former smoker, or active smoker), drinking (abstainer or active drinker) status, total energy intake and total fat intake. CI, confidence interval; PIR, poverty-income ratio, OR, odds ratio; BMI, body mass index.

**Supplemental Table 5. Estimated cutoff thresholds for the investigated p-DCB that are relevant to MetS**

| **Chemicals** | **Min-max** | **Median (IQR)** | **Thresholds*** |  |
| --- | --- | --- | --- | --- |
|  |  |  | **MetS** |  |
|  |  |  |  |  |
| **2,5-dichlorophenol (µg/L)** | 0.07-47200 | 5.5 (1.5-25.2) | 4.48 |  |
| **2,4-dichlorophenol (µg/L)** | 0.07-1260 | 0.7 (0.3-1.8) | 0.45 |  |

*Models were adjusted for: creatinine concentration, age (years), gender (male or female), race (non-Hispanic Black, non-Hispanic White, Mexican American, or others), education (under high school, high school, or above high school), PIR (< 1.52, 1.52 to 3.48, or > 3.48), physical activity (never, moderate, or vigorous), smoking (non-smoker, former smoker, or active smoker), drinking (abstainer or active drinker) status, total energy intake and total fat intake.
